# Polystyrene-Poly(methyl methacrylate) Silver Nanocomposites: Significant Modification of the Thermal and Electrical Properties by Microwave Irradiation

**DOI:** 10.3390/ma9060458

**Published:** 2016-06-13

**Authors:** Edreese H. Alsharaeh

**Affiliations:** College of Science and General Studies, Alfaisal University, P.O. Box 50927, Riyadh 11533, Saudi Arabia; ealsharaeh@alfaisal.edu; Tel.: +966-1-215-7739

**Keywords:** nanocomposites, silver nanoparticles, microwave irradiation, polymer, X-ray diffraction

## Abstract

This work compares the preparation of nanocomposites of polystyrene (PS), poly(methyl methacrylate) (PMMA), and PSMMA co-polymer containing silver nanoparticles (AgNPs) using *in situ* bulk polymerization with and without microwave irradiation (MWI). The AgNPs prepared were embedded within the polymer matrix. A modification in the thermal stability of the PS/Ag, PMMA/Ag, and PSMMA/Ag nanocomposites using MWI and *in situ* was observed compared with that of neat PSMMA, PS, and PMMA. In particular, PS/Ag, and PSMMA/Ag nanocomposites used *in situ* showed better thermal stability than MWI, while PMMA/Ag nanocomposites showed improved thermal stability. The electrical conductivity of the PS/Ag, PMMA/Ag, and PSMMA/Ag composites prepared by MWI revealed a percolation behavior when 20% AgNPs were used as a filler, and the conductivity of the nanocomposites increased to 103 S/cm, 33 S/cm, and 40 mS/cm, respectively. This enhancement might be due to the good dispersion of the AgNPs within the polymer matrix, which increased the interfacial interaction between the polymer and AgNPs. The polymer/Ag nanocomposites developed with tunable thermal and electrical properties could be used as conductive materials for electronic device applications.

## 1. Introduction

Recently, nano- and thin-film technologies based on novel systems associating metal particles with a polymer matrix have achieved unique physical and mechanical properties that were not possible with the addition of micron-sized particles. These metal nanoparticles (NPs) embedded in host polymer matrices have become the focus of increasing attention because of their applications in many fields including optical fibers, sub-wavelength waveguides, nonlinear optical switches [1], superlenses [2], magnetooptic data storages, directional connectors, electronics as nanowires [3] for biomedical materials, NP barcode labels [4,5], sensitive materials for DNA screening [6], biosensors [7], and bio-films with anti-microbial effects of Ag [8] materials. Ag, Au, and Cu NPs are reported to exhibit a strong biocidal effect on more than 16 species of bacteria including *Escherichia coli* [9,10,11]. In addition, these polymers are attractive materials for application in biosensors because of the considerable flexibility in their chemical structures and their redox characteristics [12]. The extent of modification of the property depends on the base polymer; size, distribution, and dispersion of the NPs; and adhesion at the filler–matrix interface [13]. When the NPs are embedded or encapsulated in a polymer, the polymer acts as a surface capping agent. In addition, casting of film becomes easier, and the particle size is controlled well within the desired regime. However, the key problems in this area involve the synthesis and functionalization of the NPs and their dispersion in a polymer matrix. Ag and Au are favorite NP-coating materials because of their well-known property of exhibiting optical absorption (plasmons) in the visible region. Ag has been widely studied because it is more reactive than Au. Polymers are usually flexible, lightweight, and able to provide required immobilization of the NPs, avoiding their coalescence or segregation, thus protecting the novel size-dependent properties of the nanomaterials. However, depending on the polymer as well as the concentration of the NPs, the properties of nanocomposites may change. Among various polymers, poly(methyl methacrylate) (PMMA) is a highly transparent plastic with good mechanical strength and is used variously for optical and medical applications [14,15]. In addition, polystyrene (PS) exhibits many admirable properties such as biocompatibility, nontoxicity, high surface area, strong adsorption ability, and chemical inertness [16,17,18]. Additionally, the co-polymer of methyl methacrylate and styrene (PSMMA) is an important polymeric material that has numerous applications in medicine (e.g., as bone cement); dentistry (e.g., dentures); and the paper, paint, and automotive industries [19,20]. There have been many reports on AgNPs in polymers using different techniques [21,22] including solution mixing, melt blending, *in situ* polymerization, and *in situ* polymerization using microwave irradiation (MWI) [23,24]. However, it is extremely difficult to homogenously disperse NPs into the polymer matrix because of the easy agglomeration of the NPs and the high viscosity of the polymer. The MWI method offers a fast and easy way to synthesize polymer/AgNPs materials. In MWI, dielectric heating energy is transferred directly to the reactants. Energy is supplied to the molecules faster than they are able to relax, which creates high instantaneous temperatures and increases the yield and quality of the products [25,26,27,28].

Percolation concepts are used to describe an abrupt transition from one behavior to another caused by the formation of long-range networks and have been used to describe the concentration-dependent insulator-to-conductor transition in composites of conductive fillers in insulating matrices, particularly polymer nanocomposites. These composites are technologically useful because they combine the easy processability of the polymer matrix with the desirable electrical conductivity of the filler network. Such nanocomposites could find applications in static-discharge housing and packaging for electronics; electromagnetic interference shielding; and lightweight, flexible conductors for electrodes, circuits, displays, and sensors [29].

There have been numerous reports on the usage of AgNPs as conducting-filler-based polymer composites, where improved thermal, mechanical, and electrical properties of the composites were achieved [30,31,32]. White *et al.* [32] reported the electrical percolation behavior in Ag nanowire–PS composites. These researchers showed that the conductivity of the composites can be easily tuned by modifying the aspect ratio of nanowires, and Ag nanowires are superior to carbon nanotubes because they are straight, sufficiently large that minimal interparticle variation in electrical conductivity is achieved, and disperse easily into experimental solvents and polymers. In another report, Lee *et al.* [31] prepared PMMA/polyaniline (PANI)/Ag composites using an electroless coating of Ag on a PMMA sphere pre-coated with PANI using *in situ* chemical polymerization. These researchers reported that the resistivity of the PMMA/Ag and PMMA/PANI/Ag composites varied between 10^14^ and 10^−1^ Ω·cm and 10^8^ and 10^−4^ Ω·cm, respectively.

Despite the numerous reports on composites of PS with PMMA or AgNPs, very few studies have been performed on the combination of these three components. However, the development of polymer–AgNPs nanocomposites that can fully utilize conducting filler properties and achieve significantly enhanced electrical and thermal properties with low AgNPs loading remains necessary.

In this paper, PSMMA/AgNPs nanocomposites were synthesized with a low concentration (20%) loading of AgNPs via *in situ* bulk polymerization using MWI. To the best of our knowledge, this report is the first on the enhancement of the thermal and electrical properties of PSMMA/Ag nanocomposites with percolation behavior using only 20% AgNPs filler, which exhibits a higher conductivity. Combining the advantages of PS, PMMA, and AgNPs, the nanocomposites exhibited many excellent properties, such as good solubility and dispersibility in water, satisfactory biocompatibility, and high electrical conductivity. The synthesized nanocomposites were characterized using Fourier-transform infrared spectroscopy (FTIR), ultraviolet/visible (UV/Vis) spectroscopy, X-ray photoelectron spectroscopy (XPS), Raman spectroscopy, X-ray diffraction (XRD), scanning electron microscopy (SEM), high-resolution transmission electron microscopy (HRTEM), differential scanning calorimetry (DSC), thermal gravimetric analysis (TGA), and electrical conductivity measurements to provide an understanding of the structure–property relationships as well as the percolation threshold behavior.

## 2. Results and Discussion

**Figure 1:**
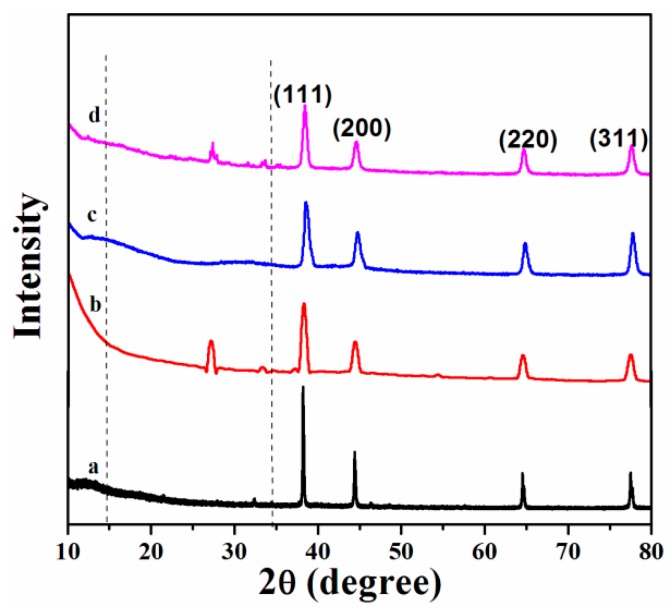
XRD patterns of (**a**) AgNPs and (**b**) PS/AgNPs; (**c**) PMMA/AgNPs; and (**d**) PSMMA/AgNPs nanocomposites.

The size and structure of the AgNPs in the polymer matrix were investigated using XRD studies; therefore, XRD patterns of the polymer/Ag nanocomposites were also obtained. Figure 1 presents XRD patterns of the AgNPs and polymer/Ag nanocomposites. As observed in Figure 1a, the pure AgNPs exhibit a crystalline nature with FCC structure with peaks corresponding to (111), (200), (220), and (311) planes. These results are consistent with the previous literature values for AgNPs and JCPDS No. 00-003-0921. Figure 1b–d presents XRD patterns of the polymer/AgNPs composites, which exhibit a two-phase (crystalline and amorphous) structure. The polymer/AgNPs exhibit the broad reflection and typical amorphous nature for the polymer, as expected, and the typical pattern of the face FCC Ag crystalline structure indicates the formation of metallic Ag.

Furthermore, the width of the (111) peak was employed to calculate the average crystallite size using the Scherrer equation [33]:

*D* = 0.9λ/(*B* × cosθ) (1)

where λ is the wavelength of the incident Cu Kα X-ray (1.514 Å), B is the full width at half maxima (FWHM) of the diffraction peak, and θ is the diffraction angle. The calculated average sizes for the AgNPs and polymer/AgNPs were observed to be ~46 nm and 18, 16, and 13 nm for PS/AgNPs, PMMA/AgNPs, and PSMMA/AgNPs, respectively. The particle size was observed to be smaller for the PSMMA/AgNPs nanocomposites than for the AgNPs. However, for the different polymer composites, the particle size did not vary greatly. The decrease in intensity and broadening of peaks in the AgNPs/polymers (Figure 1b–d) reflects the decrease in particle size of the polymer/AgNPs compared with that of the AgNPs (Figure 1a). The particle size calculated by XRD was further confirmed by TEM analysis.

**Figure 2:**
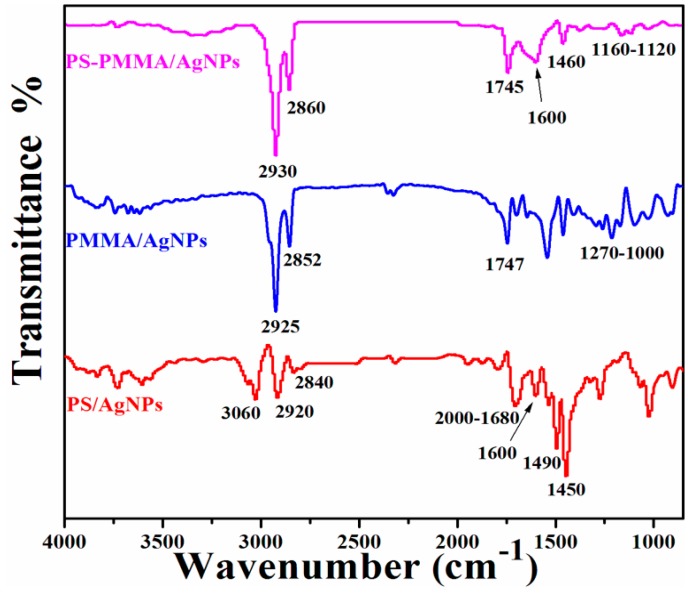
FTIR spectra of polymer/AgNPs nanocomposites.

To confirm the chemical structure of all the polymer/Ag composites, FTIR spectral analysis was performed. Figure 2 presents FTIR spectra of the PS/Ag, PMMA/Ag, and PSMMA/Ag nanocomposites. For the PS/Ag nanocomposites, the spectrum shows the presence of the characteristic bands of PS at 3060, 2920, and 2840 cm^−1^, which correspond to the aromatic ring and aliphatic C–H and –CH_2_ stretching, respectively. The aromatic overtones are observed at 1680–2000 cm^−1^, and aromatic C=C stretching is observed at 1613 cm^−1^. For the PMMA/AgNPs, the spectrum shows characteristic bands of the aliphatic C–H and –CH_2_ at 2925 and 2852 cm^−1^, respectively; the bands were reduced in intensity and became broader compared with the neat PMMA (Figure S1). The bands at 1270–1000 cm^−1^ originated from the C–H deformations and C–O–C and C–O stretching. The band at 1747 cm^−1^ is assigned to the C=O stretching vibrations of the ester group of the PMMA. It was observed that this band shifted more in the nanocomposites than the peak previously reported for neat PMMA at 1730 cm^−1^ [24]. For the PSMMA/Ag nanocomposites, the FTIR spectrum shows the typical characteristic bands at 2930 and 2860 and 1600 and 1460 cm^−1^ which correspond to the aliphatic C–H and –CH_2_ and aromatic C=C stretching, respectively, in the PS molecules. In contrast, characteristic bands at 1745 and 1160–1120 cm^−1^, which correspond to C=O stretching vibrations of ester carbonyl and C–O–C stretching vibrations, respectively, appear for the PMMA molecules [17]. Notably, the FTIR results of the polymer/Ag nanocomposites (Figure 2) demonstrate that some characteristic peaks in the aromatic of PS and ester of PMMA regions are shifted to much higher wave numbers compared with neat polymers (Figure S1). This finding may suggest that this shift is due to π–π stacking and acrylate interactions, which may be attributed to the contribution toward the stabilization of the AgNPs metal surface. These FTIR results may suggest that the π–π bonds of PS and acrylate of PMMA were opened by the MWI, which will induce more electron chain transfer sites and will thus promote more interactions between polymers and Ag.

**Figure 3:**
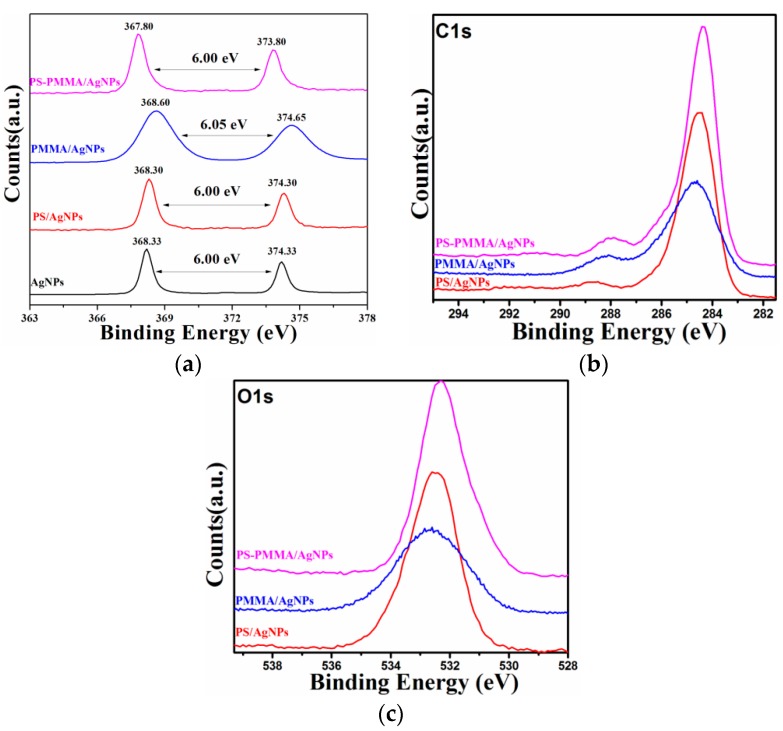
(**a**) Ag 3d XPS spectra of AgNPs; (**b**) C 1s XPS; and (**c**) O 1s spectra of polymer/AgNPs nanocomposites.

Further study on the formation of AgNPs on the surface of nanocomposites was performed using XPS, and the results are presented in Figure 3. XPS is a powerful and reliable technique for exploring the interaction of Ag NPs and polymers. The XPS survey spectra of the polymer/AgNPs (not shown here) indicated that not only Ag, O, and C were present. Detailed scans of Ag 3d, C 1s, and O 1s are presented in Figure 3a–c, respectively. In Figure 3a, the peak observed in the energy region of the Ag 3d transition is symmetrical, and two characteristic binding energy peaks for Ag 3d for metallic Ag at 374.33 and 368.33 eV are observed, corresponding to doublets of Ag 3d_3/2_ and Ag 3d_5/2_ [34], respectively. These results indicate the metallic nature of Ag, and no evidence for the existence of Ag^+^ was obtained. Therefore, the XPS study confirmed the success of the formation of metallic AgNPs within the PS/Ag, PMMA/Ag, and PSMMA/Ag nanocomposites.

Notably, for the PSMMA/Ag nanocomposites (Figure 3), the extent of two binding energies corresponding to doublet Ag 3d_3/2_ and Ag 3d_5/2_ were shifted to the lower side compared with the characteristic peaks of metallic Ag. This lower side shift (~0.05 eV) is possibly due to the stronger interaction between Ag and O in C=O groups promoted using MWI by enhancing electron transfer from the electron-rich acrylates of PMMA groups to AgNPs.

Figure 3b,c presents the C 1s and O 1s spectra of the Ag/polymer system. The peaks are wide and symmetrical. For PS/AgNPs, three different carbon functionalities are considered: (1) hydrocarbon (C–H/C–C) at 284.6 eV; (2) alcohol or ether (C–OH/C–O–C) at 285.9 eV; and (3) ester (O–C=O) at 288.6 eV [35,36], mainly due to the presence of the carbon atoms in C12 H25 SH introduced onto the AgNPs. For the PMMA/AgNPs and PSMMA/AgNPs, the profile of the C 1s line was altered, indicating that there was a change in the intensity of its three components, as observed in Figure 3b,c. In addition, the binding energy was observed to shift toward the lower side for all three components of the carbon peaks. This lower side shift in the binding energy occurred because Ag tends to lose unpaired valence electrons; therefore, a strong A–C interaction would result in a shift of the C 1s peak toward low binding energy. This claim was further confirmed by considering the O 1s spectra (Figure 3c). The O 1s core-level peaks of the polymer/AgNPs are presented in Figure 3c. As resolved by deconvolution, the O 1s spectrum consisted of a BE peak at 532.6 eV related to the O–C=O group [37,38]. This result implies interaction between Ag and O, especially between Ag and O–C=O groups. A shift in the binding energy to the lower side was observed for the PMMA/AgNPs and PSMMA/AgNPs nanocomposites, and the electron transfer from the Ag to the oxygen led to a shifting in the O 1s binding energy. The reaction between Ag and O and even polymers should be responsible for the variation.

**Figure 4:**
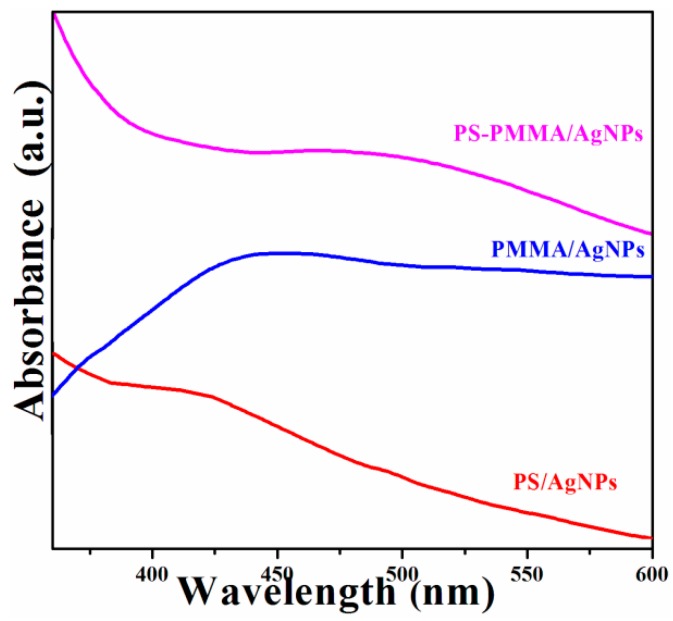
UV spectra of polymer/AgNPs nanocomposites.

The formation of AgNPs in the polymer matrix was also confirmed by UV-Vis spectra of the PS/Ag, PMMA/Ag, and PSMMA/Ag nanocomposites (Figure 4). The weak absorption band of the AgNPs at approximately 410–420 nm corresponds to the characteristic peak of metallic silver [39,40,41]. The spectra clearly demonstrate that the absorption peaks of PSMMA/AgNPs nanocomposites were red-shifted to higher wavelength compared with the PMMA/AgNPs and PS/AgNPs nanocomposites, respectively. This finding indicates the restoration of the electronic conjugation within the polymer matrix and the formation of AgNPs [31]. A very weak peak characterized the absorption peak of the AgNPs dispersion into the polymer matrix, which might be attributed to a non-aggregated dispersion of NPs [42].

**Figure 5:**
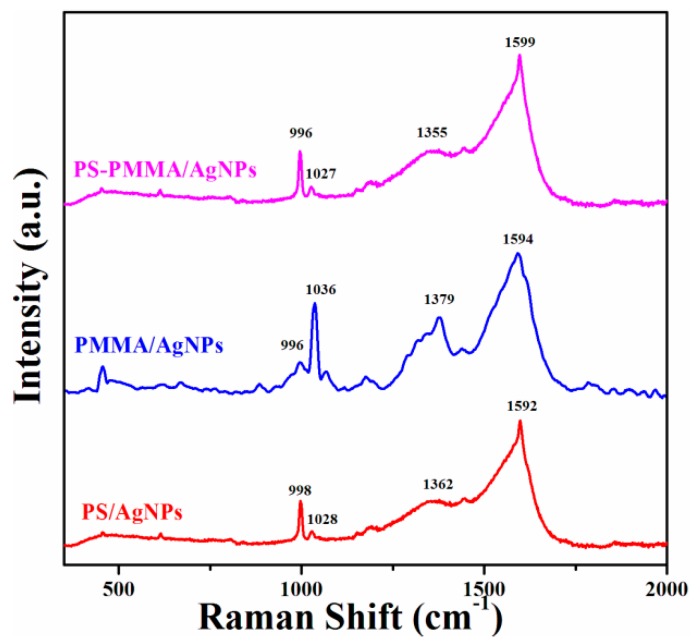
Raman spectra of polymer/AgNPs nanocomposites.

The compositions of the polymer/AgNPs nanocomposites were further examined using Raman spectroscopy (Figure 5). Raman spectroscopy is a powerful tool that provides essential information for evaluating the covalent modification of composites. The Raman spectra of the neat polymers (Figure S2) reveal the major scattering peaks of PS and PMMA. The PS spectrum contains peaks at 1602 and 1585 cm^−1^ due to stretching of benzene rings. The PMMA spectrum (Figure S2) shows characteristic peaks at 600 and 812 cm^−1^ due to stretching of C–C–O and C–COO as well as C–O–C, respectively, at 1450 cm^−1^ due to in-plane bending of C–H and at 1728 cm^−1^ due to stretching of C=O [43]. The most prominent peak appearing at 2951 cm^−1^ is due to the C–H stretching vibration. Notably, the locations of the characteristics peaks of all the polymer/Ag nanocomposites (Figure 5) were blue-shifted, which might indicate interfacial interaction between the AgNPs and the polymer matrix.

**Figure 6:**
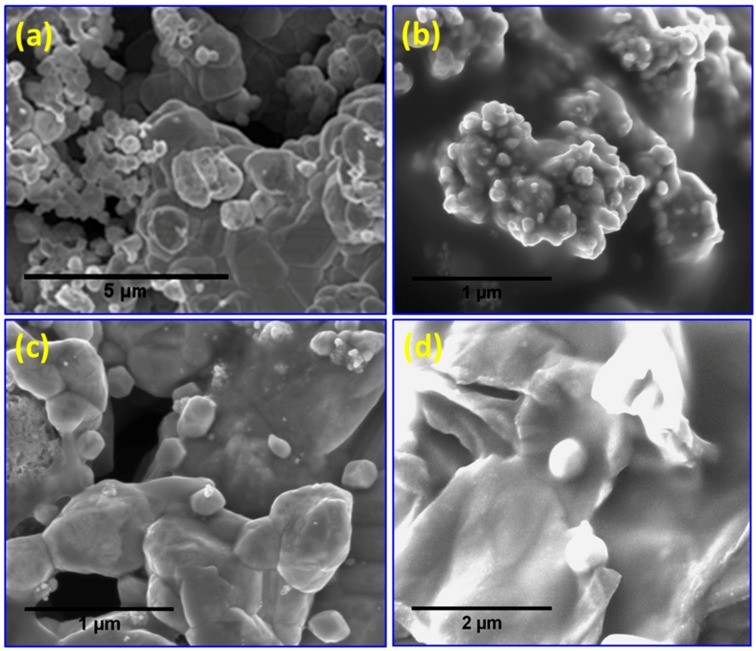
SEM micrographs of (**a**) AgNPs and (**b**) PS/AgNPs; (**c**) PMMA/AgNPs; and (**d**) PSMMA/AgNPs nanocomposites.

**Figure 7:**
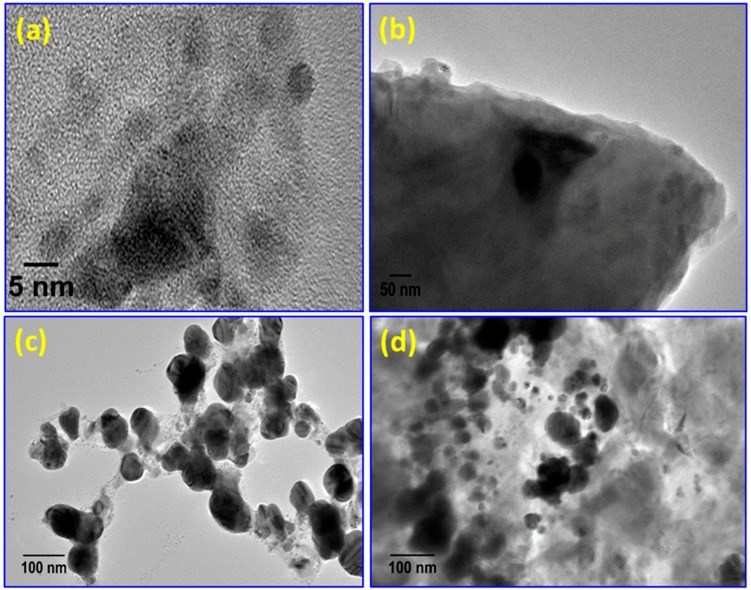
HRTEM micrographs of (**a**) AgNPs and (**b**) PS/AgNPs; (**c**) PMMA/AgNPs; and (**d**) PSMMA/AgNPs nanocomposites.

**Figure 8:**
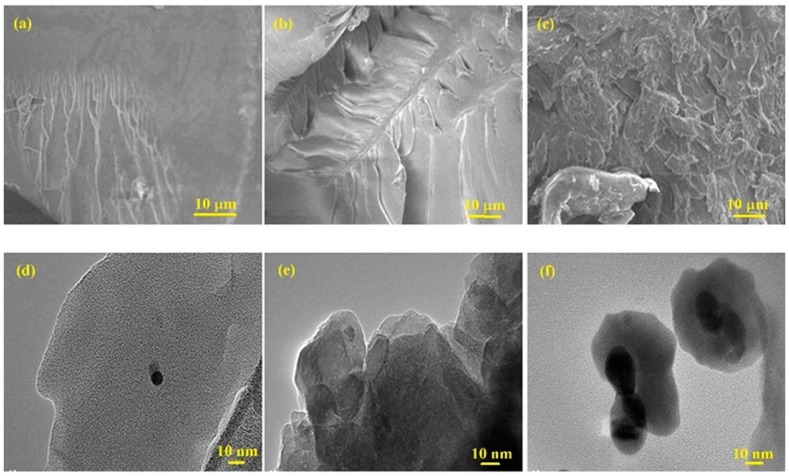
SEM and HRTEM micrographs of (**a**,**d**) PS–AgNPs; (**b**,**e**) PMMA–AgNPs; and (**c**,**f**) PS–PMMA–AgNPs nanocomposites (*in situ*).

The morphology of the polymer/Ag nanocomposites was studied using SEM and HRTEM, and the results are presented in Figure 6, Figure 7 and Figure 8. Various reports have shown that the incorporation of NPs into polymer resins can improve the mechanical and rheological properties or even introduce novel functionalities [44,45,46,47,48]. The important factors involved in improving these properties are the type, size, shape, and aspect ratio of the NPs; particle dispersion in polymer resins; and interfacial interaction between organic polymer resins and inorganic NPs. Figure 6a presents an SEM image of AgNPs of polyhedral shape with agglomerated morphology, indicating the high surface energy of these particles. The SEM micrograph of PS/AgNPs (Figure 6b) clearly reveals that very small sizes of Ag are dispersed at the surface and embedded within the PS matrix. The SEM micrograph of the PMMA/Ag nanocomposites (Figure 6c) shows that the polyhedral shaped AgNPs are dispersed within the PMMA matrix and are well separated from each other. For the PS–PMMA/Ag nanocomposites (Figure 6d), the AgNPs (white spots) are anchored and dispersed within the PSMMA matrix. According to the MARTINI force field, different values of the strength of interaction of ε represent levels of hydrophilicity/hydrophobicity [49]. A larger value of ε provides a more attractive interaction among highly polar groups, whereas a smaller one reflects a lower degree of hydrophobic repulsion between polar and nonpolar phases. The size and shape of the NPs can play an important role in changing the properties of nanocomposites. It has been experimentally determined that nanocomposites with smaller-sized NPs exhibit better performance than those containing larger-sized NPs [50,51,52]. In the present work, the NPs in the PSMMA/AgNPs nanocomposites were smaller compared with those of the other polymer composites, thus increasing the density at the polymer–NP interface. These results clearly suggest that the AgNPs were successfully embedded in the polymer matrix and modified the functional properties of the polymer.

The morphology of the nanocomposites and formation of AgNPs were further studied using HRTEM, and the results are displayed in Figure 7. The HRTEM images of all the polymer/Ag nanocomposites (Figure 7b–d) reveal good dispersion of AgNPs within the polymer matrices, with better homogeneity for PSMMA/Ag (Figure 7d) compared with the other nanocomposites (Figure 8b,c). The good dispersion and poor aggregation resulted from the van der Waals attraction between the particles, indicating the stabilization of AgNPs, which may be due to the electron transfer interaction between polymers and AgNPs. This finding is consistent with the XPS results and our previous work [17].

**Figure 9:**
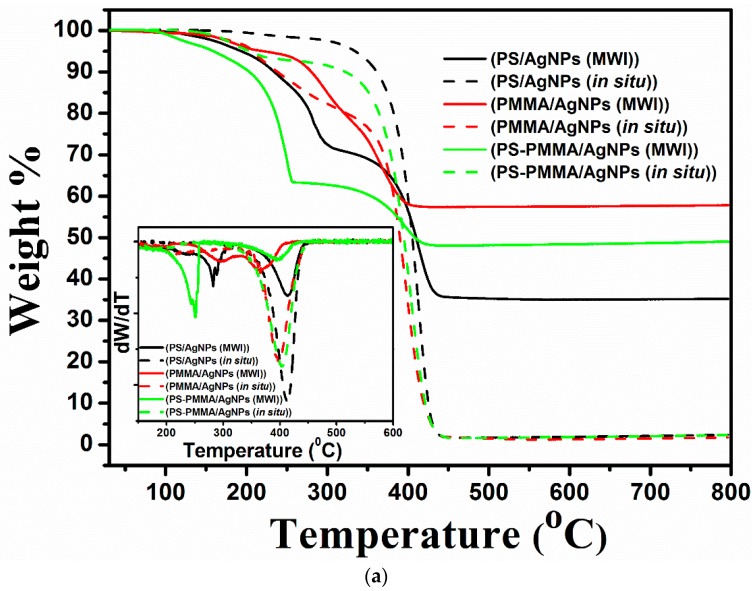

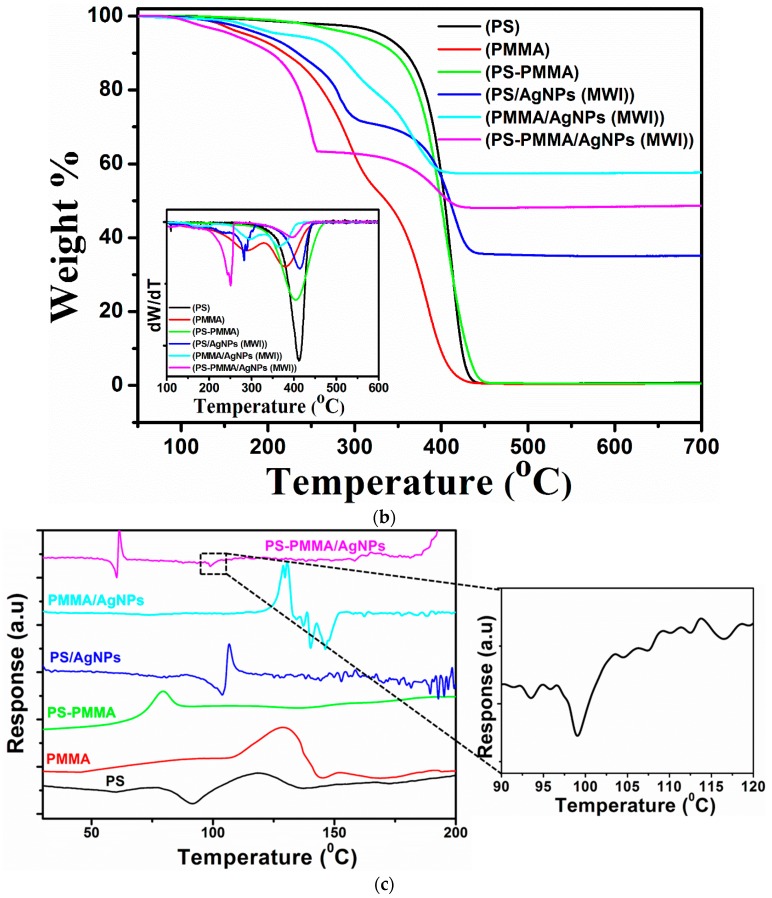
(**a**) TGA thermograms of polymer/AgNPs nanocomposites using MWI and *in situ* method; (**b**) TGA thermograms of neat polymers and polymer/AgNPs nanocomposites using MWI; (**c**) DSC thermograms of polymer/AgNPs nanocomposites and neat polymers. Bottom right: enlarged image of area marked by a box.

TGA as used to investigate the thermal stability and interfacial interaction between the AgNPs and polymer matrices. Figure 9a present the TGA curves for the polymer/AgNPs composites using MWI and *in situ* reduction, respectively; with the inclusion of AgNPs, onset degradation temperature modification was observed. The temperature at which 5% of mass loss has occurred was used for measuring the degradation temperature. TGA results show that PS/AgNPs and PS-PMMA/AgNPs nanocomposites using in-situ method showed good thermal stability than that of MWI technique. Only in the case of PMMA/AgNPs composites, those prepared using MWI have slightly better thermal stability than those prepared *in situ*. We attributed this result to the presence and dispersion of AgNPs within the polymer matrix. In MWI, dielectric heating energy is transferred directly to the reactants, and the energy is supplied to the molecules faster than they are able to relax, creating high instantaneous temperatures that increase the yield and quality of the product, which is consistent with the results from our study. Among all the polymer/AgNPs nanocomposites, PS/AgNPs prepared by the *in situ* method showed the highest thermal stability. The values of degradation are summarized in Table 1. On the basis of these results, one disadvantage of MWI method is that the composites usually have lower thermal stability.

Figure 9b shows the TGA plot of the neat polymers. The degradation of the neat polymers started at over 333, 176, and 300 for PS, PMMA, PSMMA, respectively (see Table S1). However, the PS/AgNPs (MWI) composite is less stable than pure PS; also, the composite PS-PMMA/AgNPs is less stable than pure PS-PMMA. Only in the case of PMMA/AgNPs (MWI) are the nanocomposites more stable than pure PMMA, and the thermal stability has been improved. 

Therefore, our approach is promising for the development of a new class of polymer/metal NP composites. This result demonstrates that the thermal stability of the PMMA is improved because of the presence of AgNPs using the MWI method, which is consistent with the results obtained by [53].

To understand the effect of MWI on the thermal behavior and dispersion of AgNPs within the polymer matrix, DSC of the polymer/Ag composites was performed, and the results are presented in Figure 9c and summarized in Table 1. In order to get a clear picture of the degradation and the effect of AgNPs on the degradation temperature, DSC of the neat polymers was also performed and compared, as shown in Figure 9c. For the neat polymers, the Tg values are 118 °C, 127 °C, and 79 °C for PS, PMMA, and PS-PMMA, respectively (see Table S1). For the PS/Ag nanocomposites, the thermogram shows that the *T*_g_ value of the nanocomposites (*T*_g_ = 107 °C) decreased by 11 °C compared with that of neat PS (*T*_g_ = 118 °C), as indicated in Table S1 also [54]. For the PMMA/AgNPs composite, the curve shows an improved thermal stability, with a *T*_g_ of 130 °C, which is 3 °C higher than that of the neat PMMA (*T*_g_ = 127 °C). For the PSMMA/AgNP composite, the curve shows a significantly improved thermal stability, with a *T*_g_ of 110 °C, which is 31 °C higher than that of the neat PSMMA (*T*_g_ = 79 °C). This result suggests a very strong interaction between the PSMMA chains and AgNPs. Previous work has demonstrated that the interfacial strength between nanofillers and polymers and, consequently, the thermal properties of nanocomposites can be altered by varying the sample preparation method. In this work, the *T*_g_ shift may be attributed to the presence of so-called “interphase” polymer networking, which arises because of the interaction of the chains with the AgNPs surface, which may restrict the mobility, creating an enormous volume of matrix polymer. Percolation of this network of interphase polymer could then manifest as a large *T*_g_ shift of the polymer composite. Therefore, good dispersion without agglomeration of AgNPs may result from the fast thermal reduction process that is offered by MWI for PMMA/AgNPs and PSMMA/AgNPs nanocomposites, while the *in situ* method produced more stable nanocomposites.

**Figure 10:**
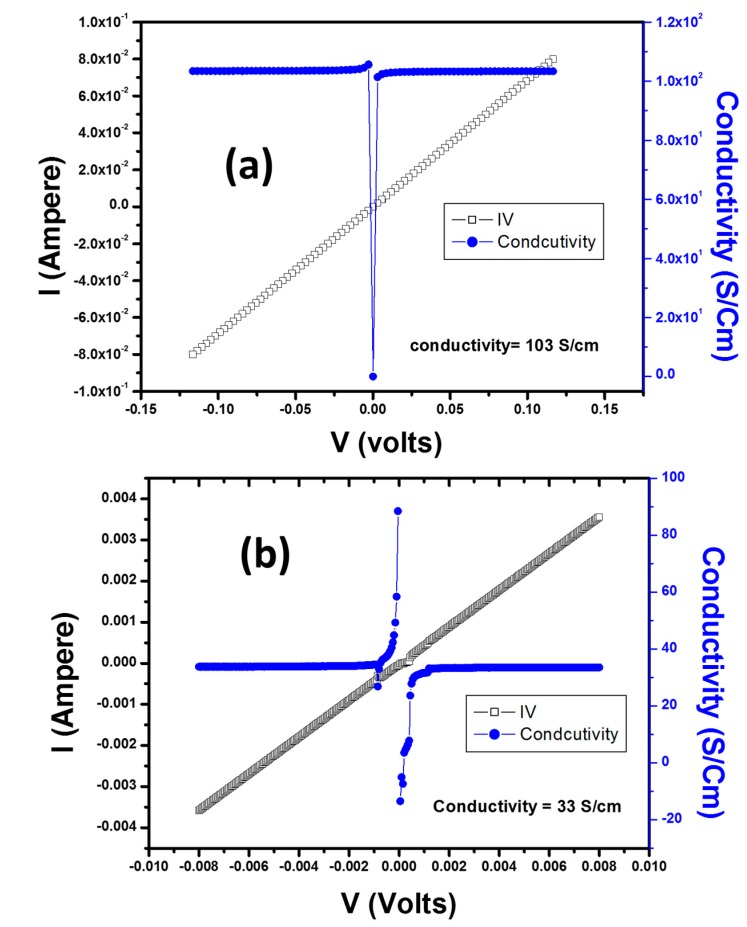

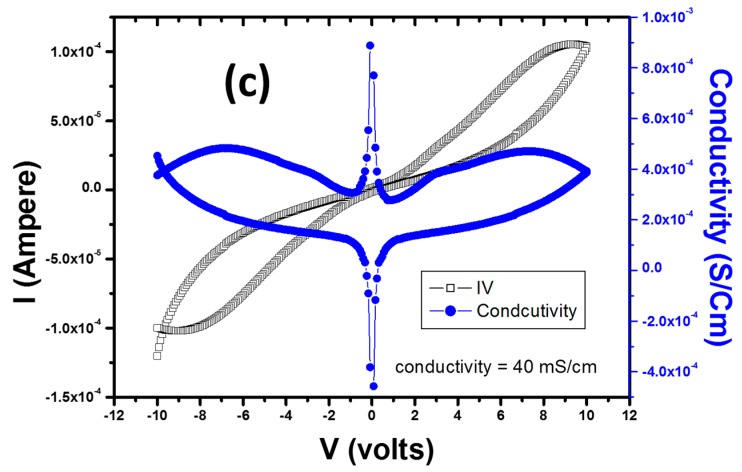
*I–V* curves of (**a**) PS/AgNP; (**b**) PMMA/Ag; and (**c**) PSMMA/Ag nanocomposites.

Pure Ag is a good conductor; it is metallic, and its conductivity on the Pauling scale is 15.87 nΩ·m at 25 °C [54] and in SI units is ~6 × 10^5^ S cm^−1^. As shown in Figure 10a, the Ps/Ag nanocomposites film exhibits a resistance of ~1.45997 Ω and a conductivity of ~103 S/cm, which is significantly lower than that of pure Ag (~6 × 10^5^ S cm^−1^). In addition, the Ps/Ag nanocomposites film exhibits an Ohmic behavior, which is clearly observed in the plot. By comparing our results with the reported work on Ps/Ag nanowires (see Table 2), we observed that the conductivity values in our work are far superior to the reported ones. White *et al.* [55] reported that the conductivity of PS/Ag nanowire composites was ~0.01 S/cm for a nanowire aspect ratio of 8, and the conductivity of Ps/Ag nanowire composites with an aspect ratio of 16 was ~100 S/cm. Further increase of the aspect ratio of (31) resulted in a conductivity of ~0.001 S/cm for PS/Ag nanowire composites.

When a polymer matrix is filled with a conducting filler, the composite gains a conductivity value of σ. When the loading of the filler is increased such that the volume filler fraction φ reaches a critical value φ_c_, an infinite cluster is formed, and the composite becomes conducting [54]. Because of the presence of a conduction or percolation path across the entire sample, a change from an insulator to a semiconductor occurs. As the filler concentration increases to the filling limit *F*, the value of *r* increases rapidly over several orders of magnitude from the value *r*_c_ at the percolation threshold to the maximal value σ_m_. Above the percolation threshold, the electrical conductivity is related to the content of conducting filler. To produce a conducting AgNPs/PMMA nanocomposites, ~16 wt % loading of AgNPs would be needed, as per the requirement of the percolation threshold. Therefore, in our case for PMMA/Ag nanocomposites, as illustrated in Figure 10b, we used 20 wt % of AgNPs, which produced conducting composites. For the percolation to occur, the volume occupied by the conducting phase in the composite is most important, which explains why the PMMA/Ag nanocomposites produced were conducting because of the percolation threshold.

For the PSMMA/Ag nanocomposites, we obtained a resistance of ~73.98347 kΩ and a conductivity of ~40 mS/cm with non-ohmic behavior, as shown in Figure 10c. These resistance and conductivity values indicate that the electrical properties of the nanocomposites were enhanced compared with that of pure PS and PMMA (see Table 1). The resistance values for pure PS and PMMA are 10^2^–10^7^ Ω and 10^14^–10^16^ Ω, respectively. To the best of our knowledge, this paper is the first to report the electrical properties of PSMMA/Ag nanocomposites. Lee *et al.* [31] reported on the effect of polyaniline on the conductivity of a PMMA/Ag hybrid composite. These researchers demonstrated that the resistivity of PMMA/Ag varied from 10^14^ to 10^7^ Ω-cm, whereas that of PMMA/PANI/Ag composites varied from 10^9^ to 10^−4^ Ω-cm. Thus, the resistivity of the PMMA/PANI/Ag composites was much lower than that of the PMMA/Ag composites, indicating that PANI strongly enhanced the conductivity. However, in our case, the resistivity is much lower than that of this work, resulting in the higher conductivity value.

## 3. Materials and Methods

### 3.1. Materials

Styrene (S) and methyl methacrylate (MMA) monomers (99%, Acros Chemical Co., One Reagent Lane, Fair Lawn, NJ, USA) were stored in a refrigerator and used as received. Benzoyl peroxide (BP) (BDH Chemicals Ltd., Dammam, Saudi Arabia) was used as an initiator, hydrazine hydrate (HH, 80%) was obtained from LobaChemi. Pvt. Ltd., Mumbai, India and silver nitrate (AgNO_3_) was obtained from Merck., Kenilworth, NJ, USA. The other solvents and chemicals were of analytical grade and used without further purification.

### 3.2. MWI Preparation of PS/Ag, PMMA/Ag, and PS–PMMA/Ag Nanocomposites

A mixture of 2.0 g (S-MMA) monomers, 80 mg AgNO_3_, and 0.1 g BP was sonicated for 1 h, and then, the mixture were maintained at 60 °C for 20 h to promote *in situ* free radical bulk polymerization. After the polymerization was completed, the product was poured into an excess of methanol, stirred for 15 min, and washed with methanol and hot water several times before being filtered and dried in an oven at 80 °C overnight. Then, a mixture of 0.40 g (PS-PMMA) polymer/AgNO_3_ composites dissolved into a solvent, 40 µL of HH, was sonicated for 1 h followed by reduction using MWI. The same procedure was performed with PS and PMMA.

### 3.3. In Situ Preparation of PS/Ag, PMMA/Ag, and PS–PMMA/Ag Nanocomposites

A mixture of 2.0 g (S-MMA) monomers, 80 mg AgNPs (prepared via MWI reduction of AgNO_3_), and 0.1 g BP was sonicated for 1 h, and then the mixture was maintained at 60 °C for 20 h to promote *in situ* free radical bulk polymerization. After the polymerization was completed, the product was poured into an excess of methanol, stirred for 15 min, and washed with methanol and hot water several times before being filtered and dried in an oven at 80 °C overnight. The same procedure was performed with PS and PMMA. The neat PS, PMMA, and PS-PMMA were prepared for comparison using the same procedure but without the addition of AgNPs.

### 3.4. Characterization

The FTIR spectra (Thermo Scientific, Waltham, MA, USA, Nicolet-iS10) of the nanocomposites were recorded in the range of 4000–500 cm^−1^. The UV-Vis spectra (Perkin-Elmer Lambda 35, Waltham, MA, USA) of the nanocomposites were recorded in the range of 200–800 nm. XRD analysis (Philips–Holland, Amsterdam, The Netherlands, PW 1729) of the nanocomposites was performed using Cu radiation (30 kV, 40 mA, Kα radiation (λ = 1.54430 Å)) between 2θ of 5° and 100°. The XPS measurements were performed using a SPECS GmbH X-ray photoelectron spectrometer. Before analysis, the samples were degassed under a vacuum inside the load lock for 16 h. The Raman spectra of nanocomposites were measured using a Bruker Equinox 55 FT-IR spectrometer equipped with an FRA106/S FT-Raman module and a liquid N_2_-cooled Ge detector using the 1064 nm line of a Nd:yttrium aluminum garnet laser with an output laser power of 200 mW. SEM (FEI Quanta 200, FEI, Hillsboro, OR, USA) was employed to examine the morphology of the nanocomposites after they were mounted on the nanocomposite slabs and coated with Au via a sputtering system (Polaron E6100, Bio-Rad, Herts HP2 7DX, Hemel Hempstead, UK). HRTEM (JEOL JSM-2100F, Tokyo, Japan) was performed at 200 kV. A drop of the composite dispersed in ethanol was placed on copper grids and dried for studies. TGA of the nanocomposites was performed under an N_2_ atmosphere at a heating rate of 10 °C per minute from 25 °C to 800 °C using a NETZCH 209 F1 thermogravimetric analyzer. DSC (NETZCH 204 F1) measurements were employed to estimate the glass-transition temperature (*T*_g_) of each nanocomposite. The nanocomposites were heated from –25 °C to 100 °C at a heating rate of 10 °C per min. Then, a double run was performed after cooling at a heating rate of 2 °C per min from 25 °C to 350 °C. The *T*_g_ was taken as the midpoint of the transition. The resistances of the nanocomposites were calculated using two point probe method in a two-electrode (Cu) configuration by using a Keithley 4200 SCS-four-probe electrical current-voltage (*I–V*) measurements system. The conductivities of the samples were calculated by fitting their *I–V* characteristics (10 cycles). After the measurements were taken, the mass of the film consisting of each composite was measured (for PS/AgNPs = 1 mg, PMMA/AgNPs = 2 mg, PS-PMMA/AgNPs = 4 mg), and the effective thickness of each film was calculated (for PS/AgNPs = 0.1 mm, PMMA/AgNPs = 0.2 mm, PS-PMMA/AgNPs = 0.4 mm)). This procedure yielded a value of the film conductivity from the measured resistance.

## 4. Conclusions

In conclusion, the incorporation of AgNPs within PS, PMMA, and PSMMA co-polymer matrices using *in situ* bulk polymerization and MWI was achieved, and the resulting functional properties were compared. FTIR and XPS studies confirmed the formation of metallic AgNPs within the polymer matrix. UV-Vis spectra of the PS/Ag and PMMA/Ag nanocomposites revealed a red shift with respect to the PSMMA/Ag nanocomposites, confirming the size effect of AgNPs. Raman spectra indicated that the characteristics peaks of all the polymer/Ag nanocomposites were blue-shifted with respect to the neat polymer, which indicates interfacial interactions between the AgNPs and polymer matrix. SEM and HRTEM micrographs of the polymer/AgNPs revealed that the AgNPs dispersed at the surface and embedded within the polymer matrix. TGA and DSC results revealed a modification in the thermal stability using AgNPs within the polymer matrix. These results indicate that the nanocomposites obtained using *in situ* technique exhibited better thermal stability than MWI—except PMMA/AgNPs (MWI), which showed better thermal stability than the *in situ* method. The electrical conductivity of the nanocomposites significantly improved, and the PS/Ag nanocomposites exhibited the highest conductivity, which is governed by the percolation model. These nanocomposites may prove particularly effective for the design of fuel cell electrodes, which are often made with conductive nanocomposites, or simply mats of conductive particles.

## Figures and Tables

**Table 1:** Thermal behavior data of polymer/AgNP nanocomposites obtained from TGA and DSC measurements.

Sample	*T*_deg_^a^ (MWI, °C)	*T*_deg_^a^ (*in situ*, °C)	*T*_g_^b^ (MWI, °C)
PS/AgNPs	191	334	107
PMMA/AgNPs	232	202	130
PS-PMMA/AgNPs	159	198	110

^a^*:* *T*_deg_ from TGA; ^b^*:* *T*_g_ from DSC.

**Table 2:** Resistance and conductivity of polymer/AgNP nanocomposites.

Nanocomposites	Resistance (Ohm)	Conductivity (S/cm)	References
Ag	10−5^−5^	6 × 10^5^	[52]
PS	10^2^–10^7^	10−2 to 10−7	[53]
PMMA	10^14^–10^16^	10^−14^ to 10^−16^	[53]
PS/Ag nanowires (A.R = 8)	10^2^	1 × 10^−2^	[53]
PS/Ag nanowires (A.R = 31)	10^3^	1 × 10^−3^	[53]
PS/Ag	10^−1^	1.4 × 10^−1^	[54]
PMMA/Ag	10^14^–10^7^	10^−14^–10^−7^	[55]
PANI-PMMA/Ag	10^9^–10^−4^	10^−9^–10^4^	[55]
PS/Ag	1.46	1.03 × 10^2^	Our Work
PMMA/Ag	10^−1^	3.3 × 10	Our Work
PS-PMMA/Ag	73.98 × 10^3^	40 × 10^−3^	Our Work

## Supplementary Materials

The following are available online at www.mdpi.com/1996-1944/9/6/458/s1. Figure S1: FTIR Spectra of Neat Polymers Polystyrene (PS), Poly methyl methacrylate (PMMA), and Polystyrene-Poly(methyl methacrylate) (PS/PMMA); Figure S2: Raman spectra of polymers Polystyrene (PS), Poly methyl methacrylate (PMMA), and Polystyrene-Poly(methyl methacrylate) (PS/PMMA); Table S1: Summary of the thermal behavior data obtained from TGA and DSC measurements.
