# Supplementary material for: Polystyrene-Poly(methyl methacrylate) Silver Nanocomposites: Significant Modification of the Thermal and Electrical Properties by Microwave Irradiation
Source: Materials (Basel). 2016 Jun 13;9(6):458. doi: 10.3390/ma9060458 (PMC5456822; doi:10.3390/ma9060458)
Supplement: Supplementary file 1 [file materials-09-00458-s001.pdf]

Supplementary Materials: Polystyrene-Poly(methyl methacrylate) Silver Nanocomposites: Significant Modification of the Thermal and Electrical Properties by Microwave Irradiation

Edreese H. Alsharaeh

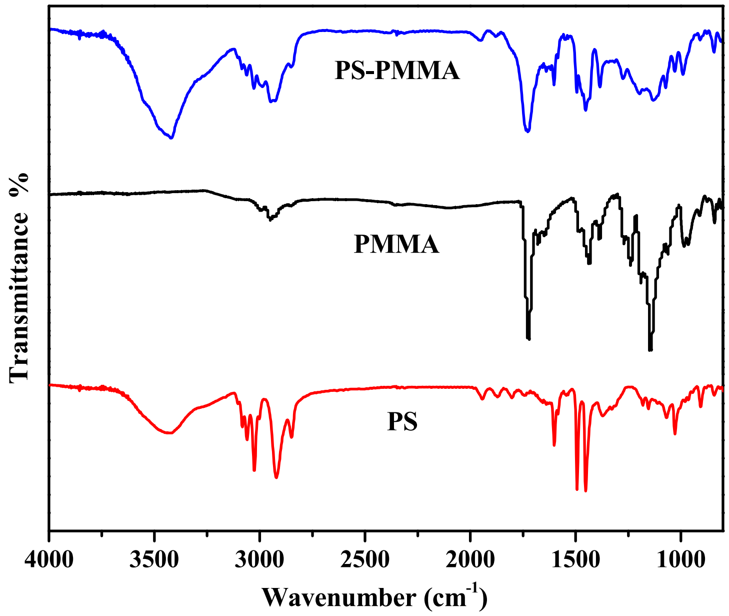

Figure S1. FTIR spectra of neat Polymers Polystyrene (PS), Poly methyl methacrylate (PMMA), and Polystyrene-Poly(methyl methacrylate) (PS/PMMA).

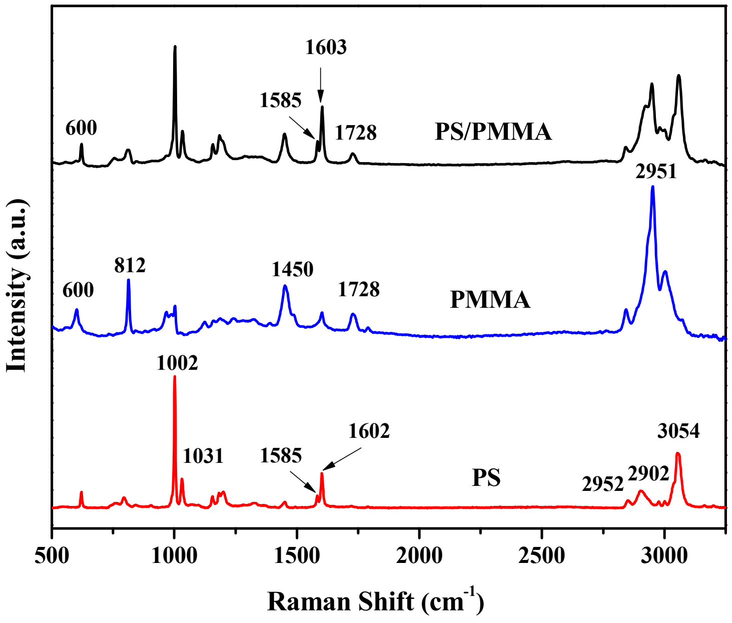

Figure S2. Raman spectra of polymers Polystyrene (PS), Poly methyl methacrylate (PMMA), and Polystyrene-Poly(methyl methacrylate) (PS/PMMA).

Table S1. Summary of the thermal behavior data obtained from TGA and DSC measurements.

**Sample**	T_{deg} [a] **(°C)**	T_g **(°C)**
PS	333	118
PMMA	176	127
PS-PMMA	300	79

[a]: The degradation temperature obtained from the DrTG (derivative thermogram) in the decomposition stage.
